# Nuciferine downregulates Per-Arnt-Sim kinase expression during its alleviation of lipogenesis and inflammation on oleic acid-induced hepatic steatosis in HepG_2_ cells

**DOI:** 10.3389/fphar.2015.00238

**Published:** 2015-10-21

**Authors:** Dan-Dan Zhang, Ji-Gang Zhang, Xin Wu, Ying Liu, Sheng-Ying Gu, Guan-Hua Zhu, Yu-Zhu Wang, Gao-Lin Liu, Xiao-Yu Li

**Affiliations:** Department of Clinical Pharmacy, Shanghai General Hospital, Shanghai Jiaotong University School of MedicineShanghai, China

**Keywords:** nuciferine, Per-Arnt-Sim kinase, non-alcoholic fatty liver disease, hepatic lipid accumulation, inflammation

## Abstract

Non-alcoholic fatty liver disease (NAFLD) is a prevalent liver disease associated with lipotoxicity, lipid peroxidation, oxidative stress, and inflammation. Nuciferine, an active ingredient extracted from the natural lotus leaf, has been reported to be effective for the prevention and treatment of NAFLD. Per-Arnt-Sim kinase (PASK) is a nutrient responsive protein kinase that regulates lipid and glucose metabolism, mitochondrial respiration, and gene expression. The aim of the present study was to investigate the protective effect of nuciferine against NAFLD and its inhibitory effect on PASK, exploring the possible underlying mechanism of nuciferine-mediated inhibition on NAFLD. Relevant biochemical parameters (lipid accumulation, extent of oxidative stress and release of inflammation cytokines) in oleic acid (OA)-induced HepG_2_ cells that mimicked steatosis *in vitro* were measured and compared with the control. It was found that nuciferine and silenced-PASK (siRNA PASK) both inhibited triglyceride (TG) accumulation and was effective in decreasing fatty acid (FFAs). The content of total antioxidant capacity (T-AOC) and superoxide dismutase (SOD) were increased respectively by nuciferine and siRNA PASK without increase in glutathione (GSH). Malondialdehyde (MDA) was decreased respectively by nuciferine and siRNA PASK. In addition, nuciferine decreased TNF-a, IL-6 and IL-8 as well as the siRNA PASK group. IL-10 was increased by nuciferine and siRNA PASK respectively. Further investigation revealed that nuciferine and siRNA PASK could respectively regulate the expression of target genes involved in lipogenesis and inflammation, suggesting that nuciferine may be a potential therapeutic treatment for NAFLD. Furthermore, the modulated effect of nuciferine on (OA)-induced HepG_2_ cells lipogenesis and inflammation, which was accompanied with PASK inhibition, was also consistent with siRNA PASK, implying that PASK might play a role in nuciferine-mediated regulation on NAFLD.

## Introduction

Non-alcoholic fatty liver disease (NAFLD) is a chronic disorder characterized by hepatic steatosis without significant alcohol consumption. NAFLD, which is believed to be associated with central obesity, dyslipidemia, hypertension, hyperglycemia, and other metabolic disorders (Tarantino et al., 2007), is accompanied with a cluster of clinic manifestations ranging from benign hepatocellular steatosis, inflammatory non-alcoholic steatohepatitis (NASH) and fibrosis to cirrhosis. According to the theory “two hits” hypothesis proposed by Day and James (1998), the pathogenesis of NAFLD is intricate. The first “hit” is deposition of free fatty acid and triglyceride in hepatocytes (steatosis), and the second “hit” is the progression of steatosis to NASH. Both progresses are accompanied with the stimulation of hepatic lipogenesis and the release of inflammation cytokines, which are the main causes of NAFLD. Therefore, several therapeutic targets have been investigated for the treatment of NAFLD, including the important mediators and nutrient sensors of cellular lipid and glucose homeostasis—AMP-activated protein kinase (AMPK; Dahlhoff et al., 2014) and peroxisome proliferator-activated receptor (PPARα/γ) (Zhao et al., 2014).

Similarly, Per-Arnt-Sim kinase (PASK), a nutrient responsive protein kinase, is an emerging regulator in lipid and glucose metabolism (Hao and Rutter, 2008; Grose and Rutter, 2010). PASK is a canonical serine/threonine kinase that contains PAS domain and can effectively regulate lipid and glucose metabolism, mitochondrial respiration, phosphorylation and gene expression (Schlafli et al., 2009; Cardon and Rutter, 2012). Hao et al. (2007) found that the metabolic rate and mitochondrial respiration were both increased in PASK knockdown mice (PASK-/-) fed with high-fat diets, and this increase was also accompanied with the increased performance of hyperactive metabolism and ATP production. To a large extent, PASK-/- could be successfully protected from obesity, implying that PASK may participate in lipogenesis and metabolism and therefore may be a new therapeutic target for the treatment of metabolic diseases and disorders such as NAFLD.

Although various medications have been developed to improve NAFLD (Vidyashankar et al., 2013; Hwang et al., 2014; Zhang et al., 2015b), their adverse effects prevent them from being more widely applied in clinical practice. Therefore, researchers have gradually focused their attention on natural products. Nuciferine is a major active ingredient extracted from the lotus leaf and has been shown to have a wide spectrum of pharmacological activities, including relaxing the smooth muscle, ameliorating hyperlipidemia, dilating vessels, preventing arrhythmia, stimulating insulin secretion, and reducing body weight (Luo et al., 2005; Ma et al., 2010; Nguyen et al., 2012). Currently, Guo et al. (2013) reported that high-fat diet-induced liver steatosis and injury in hamsters were significantly suppressed by nuciferine supplementation, indicating that nuciferine may be used as a possible remedy for NAFLD. However, the specific mechanism remains unclear. Under such circumstances, our present study aimed at exploring the protective effect of nuciferine against NAFLD and its possible underlying mechanism. HepG_2_ cells (Mersch-Sundermann et al., 2004) was selected in this study, they retain and mimic many of the specialized functions, which characterize normal human hepatocytes and used extensively to study the phase I, phase II and antioxidant enzymes ensuring that they constitute a good model to study cytoprotective, genotoxic, and antigenotoxic effects of compounds *in vitro* (Cui et al., 2010; Vidyashankar et al., 2013). Whereas, treatment with oleic acid (OA) (Araya et al., 2004) would induce morphological similarities to steatotic hepatocytes. Therefore, we established a cellular model of HepG_2_ cells treated with OA to mimic hepatic steatosis *in vitro* and explore the possible mechanism of nuciferine in attenuating lipid accumulation and inflammation. Meanwhile, based on our previous hypothesis that PASK may participant in inflammation (Zhang et al., 2015a), we silenced PASK (siRNA PASK) in HepG_2_ cells to test the possible functions and mechanism of PASK inhibition on lipid accumulation and inflammation paralleled with evaluation of nuciferine. In addition, according to human epidemiological study, vitamin E administration has been reported to be superior to placebo for the treatment of NASH in adults (Sanyal et al., 2010; Pacana and Sanyal, 2012), hence, we selected Vitamin E as our positive control paralleled with evaluation of nuciferine and siRNA PASK. The results showed that the stimulation of hepatic lipogenesis and the release of inflammation cytokines could be effectively reversed by nuciferine in OA-induced HepG_2_ cells, which was apparently consistent with the results in siRNA PASK HepG_2_ cells. More importantly, nuciferine could significantly decrease the expression of PASK, and even the relevant targets genes of PASK also showed similar trends as the alteration in siRNA PASK HepG_2_ cells. Therefore, we speculate that nuciferine and PASK could both potently modulate hepatic lipogenesis and inflammation and PASK might play a role in nuciferine-mediated regulation.

## Materials and Methods

### Materials

Cell culture medium DMEM (Gibco, USA), FBS (Gibco, European), and the antibodies (PASK, sterol regulatory element-binding protein-1c (SREBP-1c), AMPK, phospho-AMP activated protein kinase (pAMPK), nuclear factor kappa-B (NF-κB) were purchased from Abcam (USA). Oil-red-O, OA, vitamin E and nuciferine were obtained from Sigma–Aldrich (USA). Ethanol (99%), DMSO and other chemicals were purchased from Sigma–Aldrich (USA).

### Cell Culture

The human hepatocellular carcinoma cell line HepG_2_ was purchased from the Type Culture Collection of the Chinese Academy of Sciences (Shanghai, China). HepG_2_ cells were cultured with high glucose DMEM containing 10% FBS and 0.5% penicillin-streptomycin in an incubator in an atmosphere of 5% CO_2_ at 37°C with 95% humidity.

### Cytotoxicity

HepG_2_ cells were seeded in a 96-well plate at 5 × 10^3^ cells/well for 24 h, and then treated with various concentrations (0–500 μM) of nuciferine (dissolved by DMSO) and OA (40 μM) according to the Cell Counting kit-8 assay (CCK8) of a range of OA (0–1 mM, dissolved by ethanol). After 24 h incubation, absorbance readings at 490 nm were obtained using a spectrophotometer (Thermo Varioskan). Cell viability was calculated as follows: Cell viability = (A_nuciferine__+__OA_ – A_blank_)/(A_DMSO__+__ethanol_ – A_blank_) × 100%, using HepG_2_ cells without both OA and nuciferine as control.

### Cell Apoptosis

Cells treated with nuciferine (10, 25, and 50 μM) were stained with PE Annexin V and 7-Amino-Actinomycin (7-AAD) following the manufacturer’s instructions to detect early apoptotic cells (PE Annexin V+7-AAD events) and late apoptotic cells (PE Annexin V+7-AAD events). HepG_2_ cells without both OA and nuciferine were selected as control and all groups were examined by flow cytometry (BD Accuri C6).

### OA-induced Steatosis

Oleic acid was selected to induce cellular steatosis in HepG_2_ cells as previously described (Araya et al., 2004; Vidyashankar et al., 2013; Hwang et al., 2014). HepG_2_ cells seeded in a 6-well plate at 6-well plate 5 × 10^5^ cells/well, cultured for 24 h, and then added with the medium containing different concentrations of OA (40, 80, 120, 160, and 200 μM) for another 24 h. The medium without OA but containing BSA was selected as the control. The optimum dosage was evaluated by both oil-red-o staining and cell viability of each concentration.

### Staining Using Oil-Red-O

After incubation of HepG_2_ cells (5 × 10^5^ cells/well) with BSA medium containing OA (40, 80, 120, 160, and 200 μM), or cultured with BSA-OA (40 μM) medium complex with or without nuciferine and the positive control vitamin E (25 μM) for 24 h, cells were washed with PBS three times and fixed in 4% paraformaldehyde at room temperature for 40 min. After removing the fixative, cells were washed with PBS three times, stained with a freshly prepared working solution (3:2) of oil-red-O, and incubated for 15 min at room temperature. After removing the oil-red-O working solution, cells were washed with PBS five times, and finally observed under a microscope. Similar methods were used in siRNA PASK cells incubated with BSA-OA for 24h. The staining extent of oil-red-O was quantitated with Image-Pro Plus 6.0. The mean optical density (MOD) was calculated as follows: MOD = IOD/SUM Area.

### The Inhibitory Effect of Nuciferine or siRNA PASK on OA-induced Hepatic Steatosis, Inflammation and Oxidative Stress

Based on the optimal dosage of OA (40 μM) according to experiments as described earlier, HepG_2_ cells (5 × 10^5^ cells/well) were incubated with BSA-OA (40 μM) complex with or without nuciferine, and PASK-silenced (siRNA PASK) HepG_2_ cells were treated with BSA-OA (40 μM) medium, using vitamin E (25 μM) as the positive control, HepG2 cells without both OA and nuciferine as control and siRNA PASK HepG2 cells without OA as siRNA scrambled. After 24 h treatment, cell culture medium and cell protein supernatants were harvested and kept at -80°C for quantification of several parameters.

#### Measurement of Lipid Accumulation

The extent of lipid accumulation was measured by TG from cell protein supernatants and the Fatty acid (FFAs) concentration from cell culture medium. Both of them were quantitated by commercially available enzyme linked immunosorbant assay (ELISA) following the user guide (CUSABIA, China).

#### Measurement of Inflammatory Cytokines

The evaluation of inflammation cytokine including TNF-α, IL-8, IL-6, and IL-10 in the cultured medium were assayed using an ELISA kit according to the manufacturer’s instructions.

#### Measurement of Oxidative Stress

Oxidative stress was measured with the level of glutathione (GSH), the content of total antioxidant capacity (T-AOC) and superoxide dismutase (SOD) from cell protein supernatants. All of them were assayed using the ELISA kit according to the manufacturer’s protocol.

#### Measurement of Lipid Peroxidation

The level of Malondialdehyde (MDA) from cell cultured serum was related to lipid peroxidation which was quantitated by ELISA.

### Western Blots Analyses

Cells treated with nuciferine or siRNA PASK HepG_2_ cells were lysed with lysis buffer (150 nM sodium chloride, 1% Triton X-100, 1% sodium deoxycholate, 0.1% sodium dodecyl sulfate, 50 nM Tris-HCl, and 2 mM ethylenediamine tetra-acetic acid) on ice for 45 min. After centrifugation at 12000 rpm at 4°C for 15 min, the supernatant was collected, and the protein concentration was determined by BSA method. Equal amounts were separated by sodium dodecyl sulfate polyacrylamid Gel electrophoresis (SDS-PAGE) and electro-transferred onto polyvinylidene difluoride (PVDF) membranes, which were then blocked with 5% bovine serum albumin (BSA) and probed with primary antibodies against PASK, SREBP-1c, AMPK, p-AMPK, NF-κB at 4°C overnight, washed with 1% TBST (TBS containing 1% Tween-20) three times before incubation in alkaline phosphatase-conjugated secondary antibody for 1.5 h at room temperature. After three washes, the chemiluminescence signal was imaged using a ChemiDoc XRS (Bio-Rad) and quantitated using Quantity One software (Bio-Rad).

### Real-Time Quantitative Reverse Transcription-PCR Analyses (RT-qPCR)

Total RNA of cells treated with nuciferine or siRNA PASK HepG_2_ cells were extracted by using Trizol (Invitrogen) and verified by electrophoresis. The mRNA was reverse-transcribed into cDNA with a reverse-transcription kit (Thermo Fisher Scientific). The Primer sequences listed in **Table 1** used for PCR were from Sangon, Biotech. SYBR Green I (Amersco) was added into the reaction mixture. RT-qPCR was performed on an MJ Opticon 2 thermal cycler (MJ Research Inc.) following the manufacturer’s instruction.

**Table 1:** Sequences of primers.

Gene	Forward Primer (5′-3′)	Reverse Primer (5′-3′)	Size
β-actin	AGCGAGCATCCCCCAAAGTT	GGGCACGAAGGCTCATCATT	285bp
SREBP-1c	TGGCTGCTCAATGGGCTGTT	GCGATGCCTCCAGAAGTACACG	136bp
PPAR-γ	GGAGGTCCGCATCTTTCACT	GCTACCAGCATCCCGTCTTT	195bp
PPAR-α	CTGGTAGCGTATGGAAATG	AAATGATAGCAGCCACAAA	160bp
TNF-α	TCAGAGGGCCTGTACCTCAT	GGAAGACCCCTCCCAGATAG	220bp
NF-κB	CACTTAGCAATCATCCACCTT	GCAAATCCTCCACCACATCTT	163bp
SCD1	CCCAGCTGTCAAAGAGAAG	CAAGAAAGTGGCAACGAACA	187bp
PASK	AGCTGAAATCCCAACCCA	CTCCGTCTTTCCGTAACCA	185bp
AMPK	GTGACCTCAAGCCTTCCAAC	TTTCTGGAGCCCTGTACCAA	150bp
Akt	GCACCTTCCATGTGGAGACT	CCCAGCAGCTTCAGGTACTC	214bp
ACC	CATGCGGTCTATCCGTAGGT	TGTTGTTGTTTGGTCCTCCA	158bp
FAS	TGCCAAGAAGGGAAGGAG	TGGTGTTGCTGGTGAGTG	238bp

### Immunostaining

Cells treated with nuciferine or siRNA PASK HepG_2_ cells were washed three times with PBS, fixed in 4% paraformaldehyde at 37°C for 20 min, permeabilized in 0.5% Triton-X for 15 min, and blocked in 5% goat serum for 1 h. After incubation with the appropriate primary (overnight incubation at 4°C) and secondary (2 h at 37°C) antibodies, the cells were imaged using a confocal laser scanning microscope (Leica TCS SP8).

### PASK Silencing

HepG_2_ cells were transfected with human PASK-specific siRNAs (Oobio) using transfection reagent (Oobio) according to the method of Wu et al. (2014). SiRNA sequence: GCTGATGGAAAGCCAAGACAT.

### Statistical Analysis

The results are described as mean ± SEM from three independent experiments. Statistical significances were tested by the ANOVA and the Student’s *t*-test. Data analysis was carried out using GraphPad Prism version 6 (GraphPad Software). *P*-values below 0.05 were considered statistically significant.

## Results

### OA-induced TG Accumulation in HepG_2_ Cells

**FIGURE 1:**
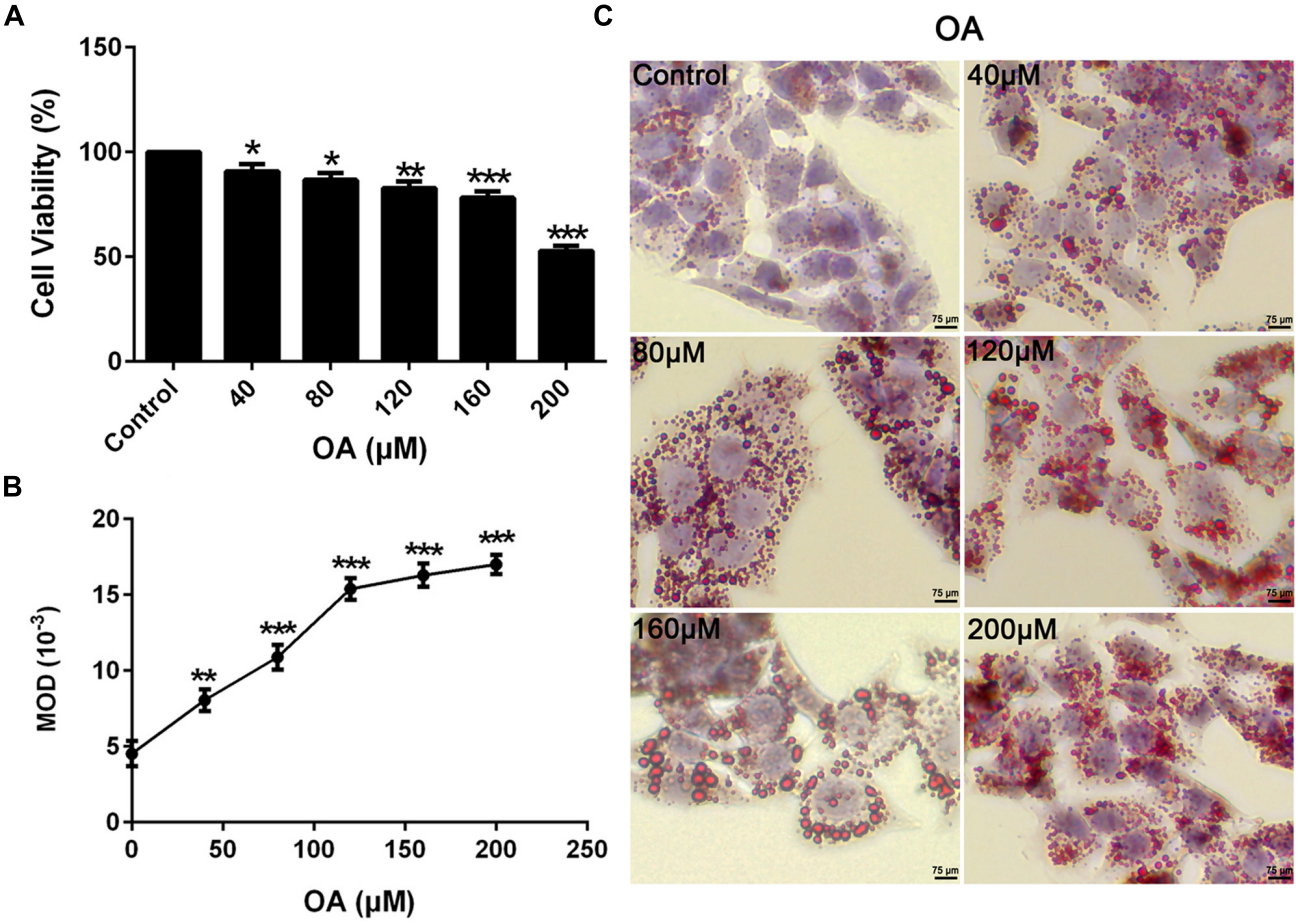
**Oleic acid-induced TG accumulation in HepG_2_ cells. (A)** The Dose dependent effect of OA (40, 80, 120, 160, and 200 μM) on cytotoxicity in HepG_2_ cells. **(B,C)** The effect of OA (40, 80, 120, 160, and 200 μM) on TG accumulation in HepG_2_ cells. Original magnification was 200×, scale bars represent 75 μm. Values are Mean ± SEM of three independent experiments performed in triplicates. Statistically significant at ^∗^*P* < 0.05, ^∗∗^*P* < 0.01 and ^∗∗∗^*P* < 0.001 compared with the control.

To mimic the hepatic steatosis *in vitro*, HepG_2_ cells were treated with 0–200 μM concentrations of OA for 24 h to induce TG steatosis. The optimal concentration depended on the cytotoxic effect of OA and the TG content evaluated by oil-red-O staining. Compare to the control (HepG_2_ cells without OA), the cell viability value ranged from 52 to 91% (**Figure 1A**) (^∗^*P* < 0.05, ^∗∗^*P* < 0.01 and ^∗∗∗^*P* < 0.001) while the recovered oil-red-O content (determined by MOD) was increased significantly (**Figures 1B,C**) (^∗∗^*P* < 0.01 and ^∗∗∗^*P* < 0.001). Cell viability values greater than 90% were considered the unaffected concentration combined with the increased TG content. Therefore, 40 μM OA was selected as the final concentration to induce TG accumulation.

### Cytotoxicity and Apoptosis of Nuciferine

**FIGURE 2:**
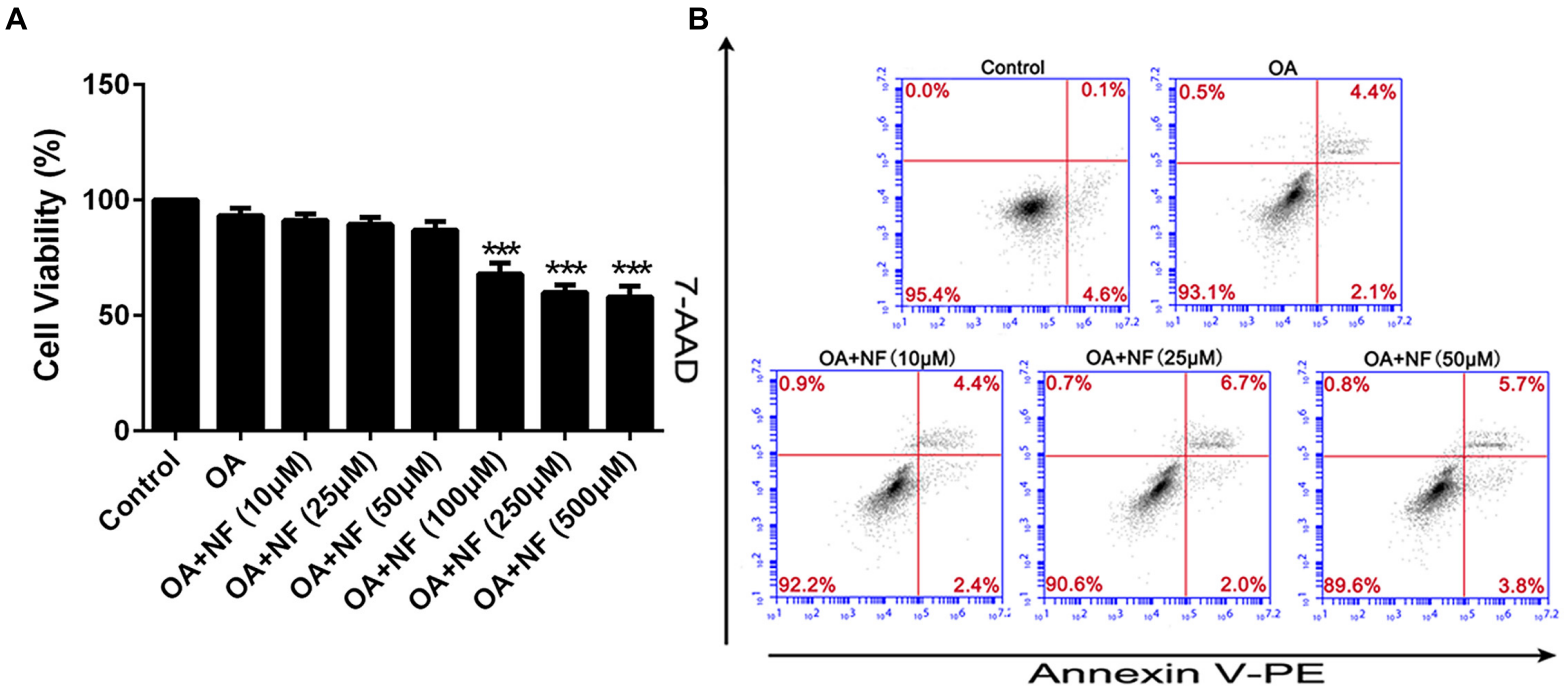
**Cytotoxicity and apoptosis of nuciferine. (A)** Cytotoxicity of nuciferine in HepG_2_ cells. HepG_2_ cells were incubated with OA (40 μM) and increased concentrations of nuciferine (10, 25, 50, 100, 250, and 500 μM) for 24 h, and cell viability was measured by CCK8 assay. **(B)** The dose dependent effect of nuciferine (10, 25, and 50 μM) on cell apoptosis in HepG_2_ cells. Values are Mean ± SEM of three independent experiments performed in triplicates. Statistically significant at ^∗∗∗^*P* < 0.001 compared with the control.

To eliminate any misinterpretation owing to cell apoptosis or death caused by toxicity of nuciferine, apoptosis, and cytotoxicity on HepG_2_ cells were assessed. Cells were treated with the OA (40 μM) and 0–500 μM nuciferine for 24 h. The cytotoxic effect of nuciferine was determined by cell viability. Compare to the control (HepG_2_ cells without both OA and nuciferine), the value ranged from 57 to 91% at 0–500 μM concentrations and values greater than 85% were considered unaffected concentrations (^∗∗∗^*P* < 0.001 vs. control). Therefore, 10 μM, 25 μM, and 50 μM nuciferine concentrations were used to reduce fatty liver (**Figure 2A**). In addition, nuciferine concentrations ranging from 10 to 50 μM did not significantly induce apoptosis of HepG_2_ cells according to flow cytometric analysis (**Figure 2B**).

### The Effect of Nuciferine or siRNA PASK on TG Accumulation in OA-induced Hepatic Steatosis

**FIGURE 3:**
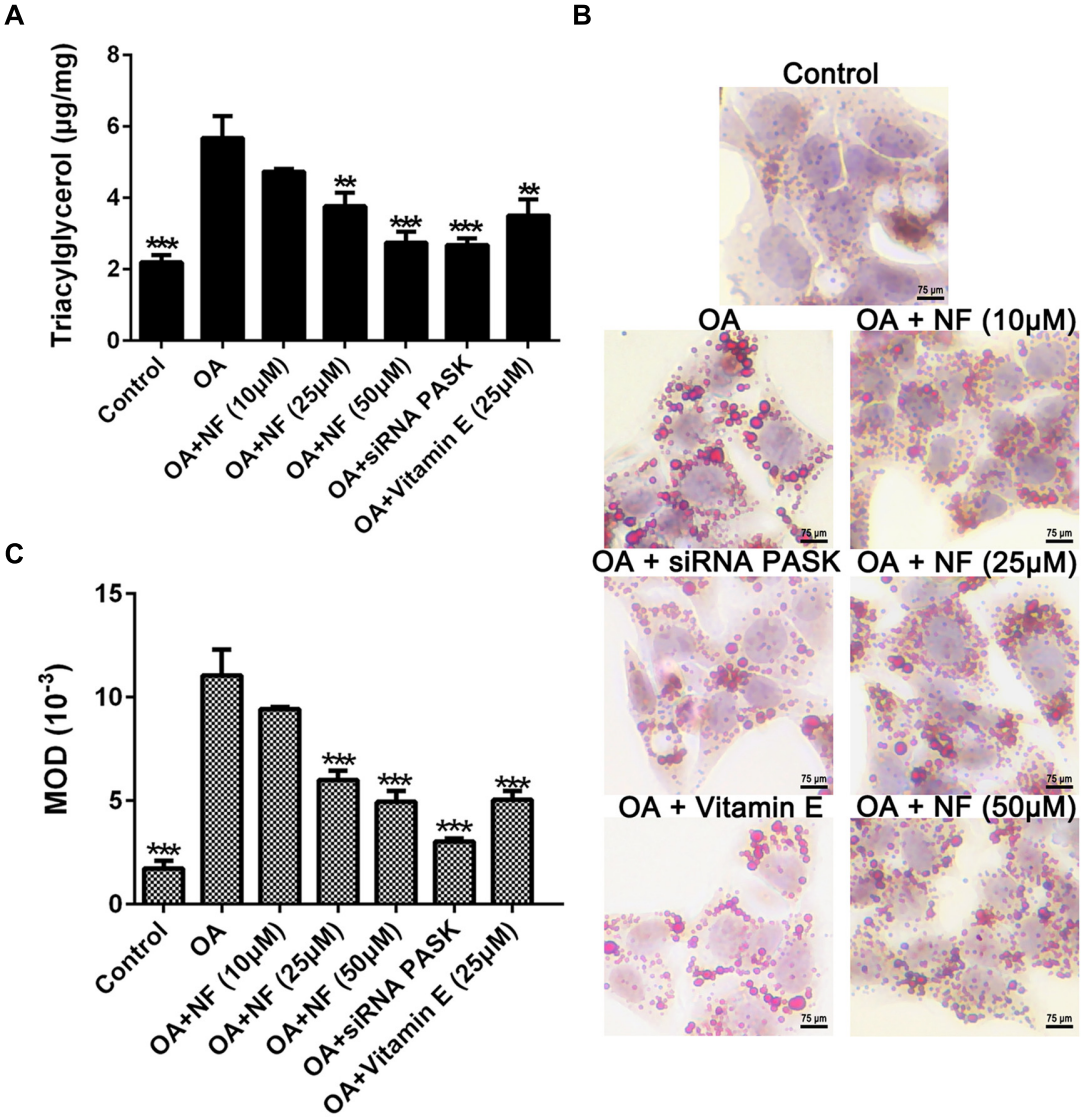
**The effect of nuciferine or siRNA PASK on TG accumulation in HepG_2_ cells of OA-induced hepatic steatosis. (A)** TG concentration tested by ELISA. **(B,C)** Oil-red-O staining. Original magnification was 200×, scale bars represent 75 μm. SiRNA PASK HepG_2_ cells and HepG_2_ cells incubated with increasing concentrations of nuciferine (NF: 10, 25, and 50 μM) or vitamin E (25 μM) both treated with OA (40 μM). The measurement was described in the Section “Materials and Methods”. Values are Mean ± SEM of three independent experiments performed in triplicates. Significant differences with OA group were designated as ^∗∗^*P* < 0.01 and ^∗∗∗^*P* < 0.001. (Control, siRNA scrambled; NF, Nuciferine.)

To examine the catabatic role of nuciferine and silenced PASK in TG accumulation, any decrease in TG content was evaluated using the ELISA kit and oil-red-O staining. Owing to the results of all parameters in the control group were almost consistent with the siRNA scrambled (data were not shown), for more concise, we selected siRNA scrambled as control in our next results. It was found that TG accumulated in OA treated cells as compared with the control (^∗∗∗^*P* < 0.001). The three concentrations of nuciferine decreased TG accumulation by 16.6, 33.7, and 52.7% respectively (^∗∗^*P* < 0.01 and ^∗∗∗^*P* < 0.001 vs. OA), while vitamin E (25 μM) and siRNA PASK reduced the TG content by 38.6% (^∗∗^*P* < 0.01 vs. OA) and 53% (^∗∗∗^*P* < 0.001 vs. OA) respectively as compared with OA group (**Figure 3A**). Microscopic examination using oil-red-O staining showed that lipid droplets in cells treated with 10, 25, and 50 μM nuciferine decreased by 15, 45.5, and 60.7% respectively as determined by MOD (^∗∗∗^*P* < 0.001 vs. OA), 54.6% for 25 μM vitamin E (^∗∗∗^*P* < 0.001 vs. OA) and 63% for siRNA PASK (^∗∗∗^*P* < 0.001 vs. OA) as compared with OA group (^∗∗∗^*P* < 0.001 vs. control) (**Figures 3B,C**). These data initially imply that both nuciferine and siRNA PASK had a regulatory effect on lipid accumulation.

### The Effect of Nuciferine or siRNA PASK on Cell Lipotoxicity in OA-induced Hepatic Steatosis

**FIGURE 4:**
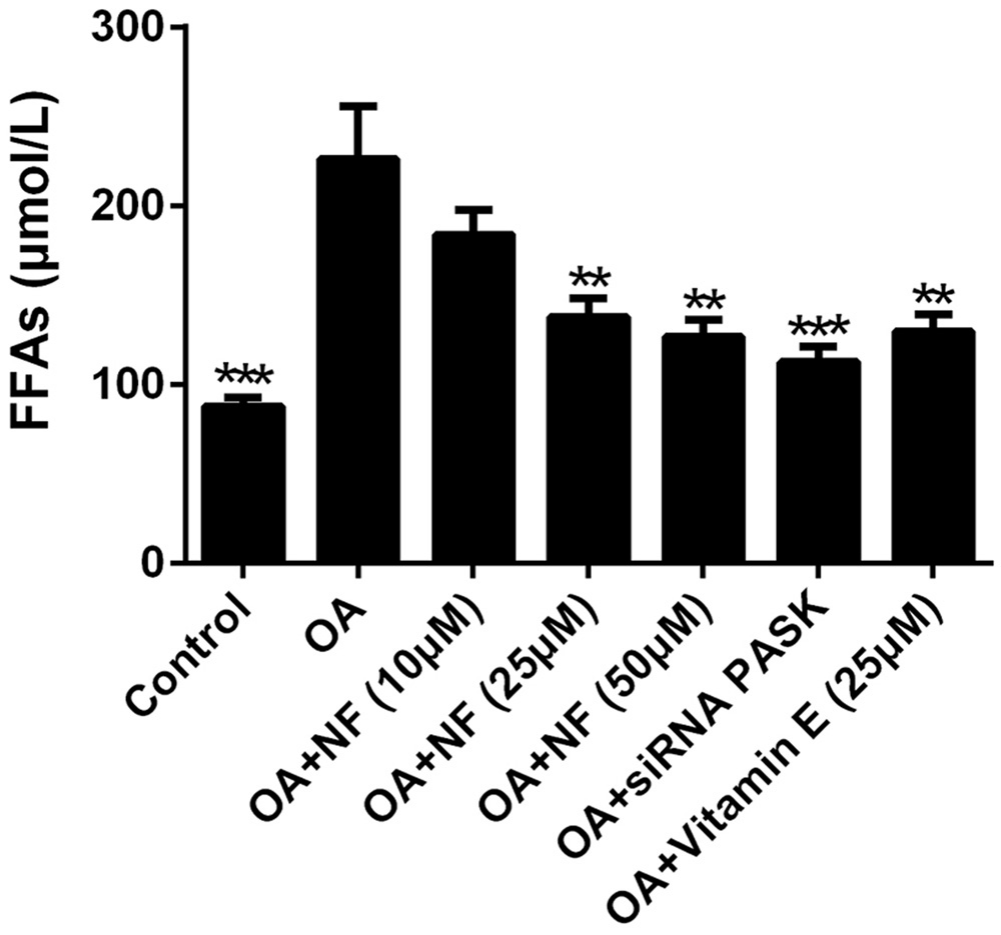
**The effect of nuciferine or siRNA PASK on cell lipotoxicity in HepG_2_ cells of OA-induced hepatic steatosis.** SiRNA PASK HepG_2_ cells and HepG_2_ cells incubated with increased concentrations of nuciferine (NF: 10, 25, and 50 μM) or vitamin E (25 μM) both treated with OA (40 μM). The measurement was described in the Section “Materials and Methods”. Values are Mean ± SEM of three independent experiments performed in triplicates. Significant differences with OA group were designated as ^∗∗^*P* < 0.01 and ^∗∗∗^*P* < 0.001. (Control, siRNA scrambled; NF, Nuciferine.)

To further explore the regulatory effect of nuciferine on lipid accumulation, the lipotoxicity caused by a cluster of FFAs that induced hepatic steatosis was measured. The addition of OA resulted in a 2.57-fold increase in FFAs as compared with the control (^∗∗∗^*P* < 0.001). The increased concentration of nuciferine decreased the FFAs level by 18.66, 39 and 44.01% respectively (^∗∗^*P* < 0.01 vs. OA), while vitamin E (25 μM) and siRNA PASK inhibited cellular FFAs by 42.81% (^∗∗^*P* < 0.01 vs. OA) and 50.22% (^∗∗∗^*P* < 0.001 vs. OA) respectively as compared with OA group (**Figure 4**). These data indicate that nuciferine and siRNA PASK played a regulatory role in attenuating lipid accumulation, similarly.

### The Effect of Nuciferine or siRNA PASK on the Expression of Lipogenic Genes Related to Hepatic Steatosis

**FIGURE 5:**
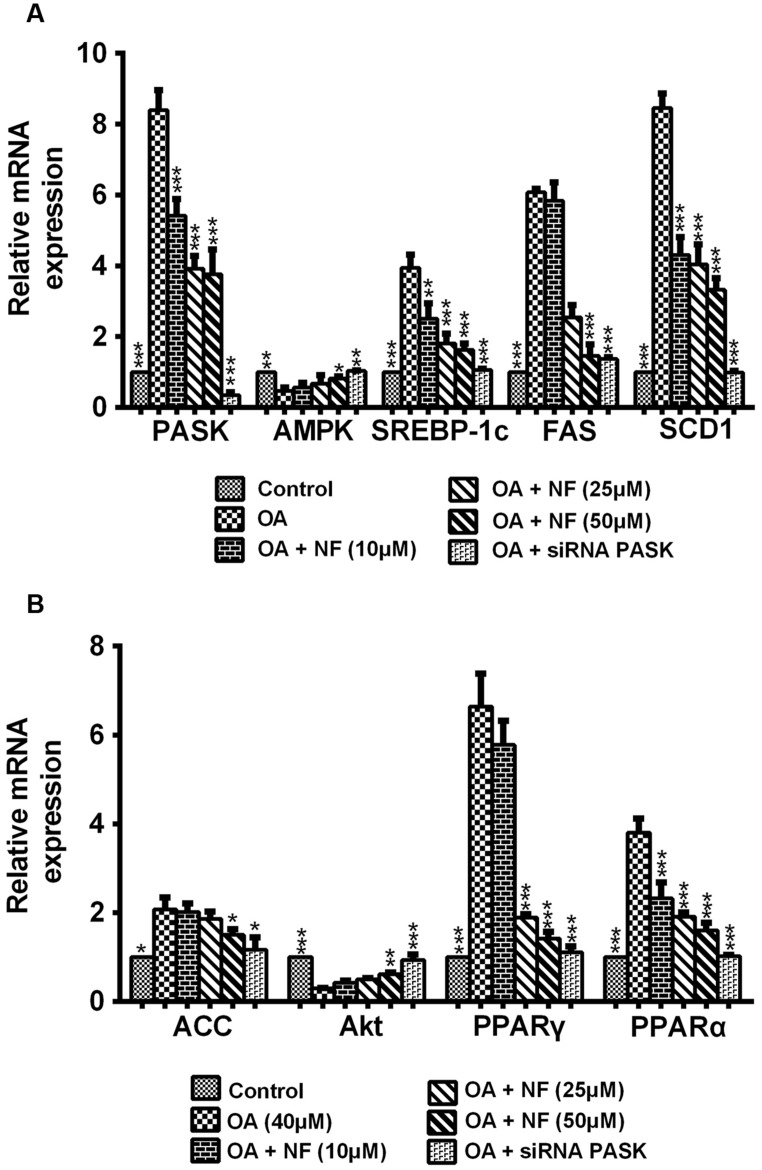
**(A,B)** The effect of nuciferine or siRNA PASK on lipogenic genes related to hepatic steatosis in HepG_2_ cells. SiRNA PASK HepG_2_ cells and HepG_2_ cells incubated with increased concentrations of nuciferine (NF: 10, 25, and 50 μM) both treated with OA (40 μM). The measurement was described in the Section “Materials and Methods”. Values are Mean ± SEM of three independent experiments performed in triplicates. Significant differences with OA group were designated as ^∗^*P* < 0.05, ^∗∗^*P* < 0.01, and ^∗∗∗^*P* < 0.001. (Control, siRNA scrambled; NF, Nuciferine.)

To decipher the possible mechanism underlying the nuciferine-mediated effect on lipogenesis, nine important genes involved in lipogenesis were evaluated. According to results of the mRNA expression by RT-qPCR, the expression of PASK, SREBP-1c, fatty acid synthase (FAS), acetyl-CoA (ACC), stearoyl-CoA desaturase 1 1 (SCD1), PPAR-α and PPAR-γ was increased in OA-treated cells compared with the control (^∗^*P* < 0.05 and ^∗∗∗^*P* < 0.001), and this increase could be significantly reversed by nuciferine (10, 25, and 50 μM) and siRNA PASK. Meanwhile, the level of AMPK and protein kinase B (Akt) showed significantly decreased in OA-induced HepG_2_ cells (^∗∗^*P* < 0.01 and ^∗∗∗^*P* < 0.001), and they both increased when treated with nuciferine and siRNA PASK (^∗^*P* < 0.05, ^∗∗^*P* < 0.01, and ^∗∗∗^*P* < 0.001 vs. OA) (**Figures 5A,B**). These findings demonstrate that nuciferine could attenuate lipid accumulation by regulating the expression of relevant target genes involved in lipogenesis, which was accompanied with downregulated expression of PASK and was consistent with alterations of relevant genes in siRNA PASK group.

### The Effect of Nuciferine or siRNA PASK on the Expression of Proteins Related to Hepatic Steatosis

**FIGURE 6:**
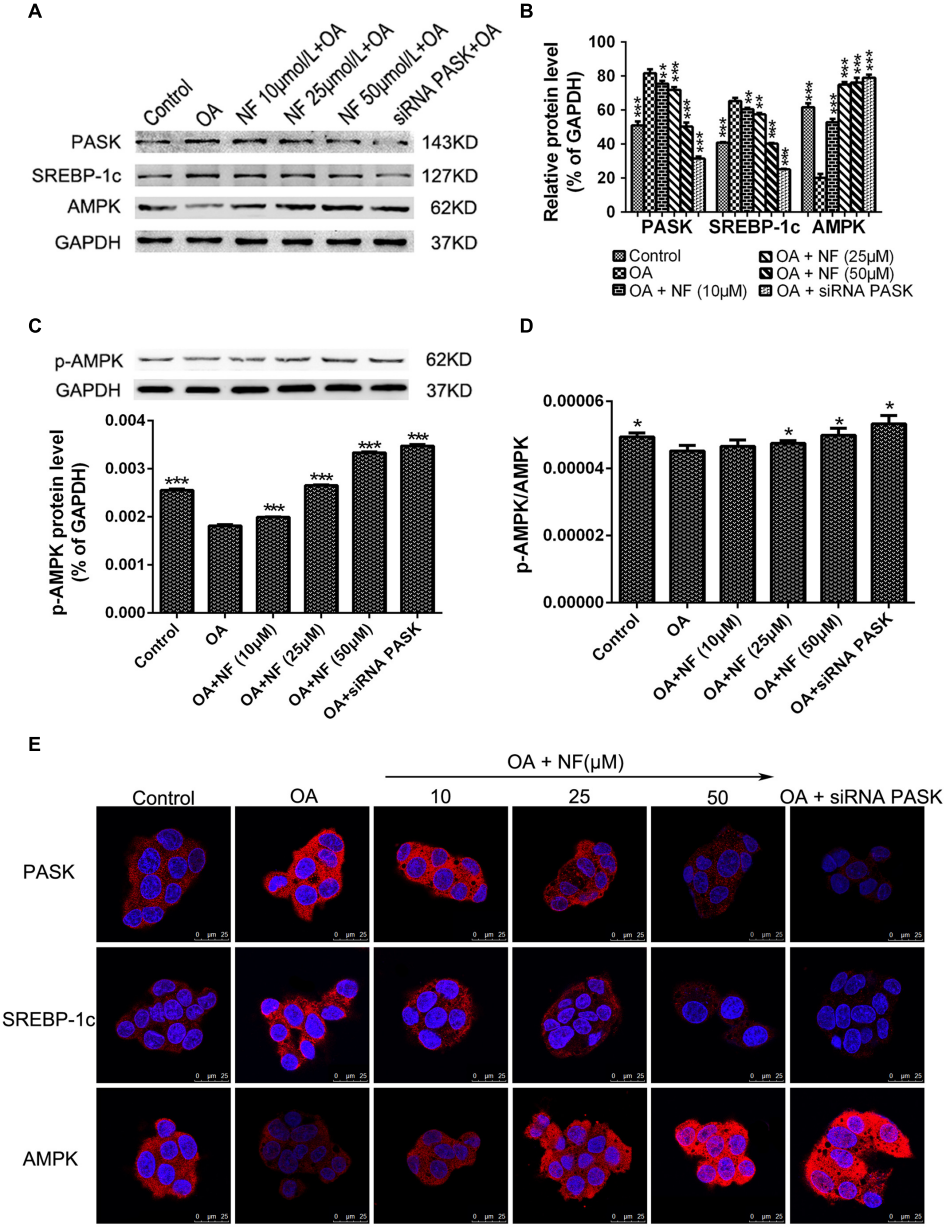
**The effect of nuciferine or siRNA PASK on the expression of proteins related to hepatic steatosis in HepG_2_ cells. (A–C)** Western blot for PASK, SREBP-1c, AMPK, and p-AMPK. **(D)** The ratio of pAMPK/AMPK (p-AMPK^∗^GAPDH(A)/GAPDH(p)^∗^AMPK). **(E)** Immunostaining for PASK, SREBP-1c and AMPK (red) and the nuclear dye DAPI (blue), scale bars represent 25 μm. SiRNA PASK HepG_2_ cells and HepG_2_ cells incubated with increasing concentrations of nuciferine (NF: 10, 25, and 50 μM) both treated with OA (40 μM). The measurement was described in the Section “Materials and Methods”. Values are Mean ± SEM of three independent experiments performed in triplicates. Significant differences with OA group were designated as ^∗∗^*P* < 0.01 and ^∗∗∗^*P* < 0.001. (Control, siRNA scrambled; NF, Nuciferine.)

To further characterize the downregulation of PASK mediated by nuciferine, western blot analysis of HepG_2_ cells from the experimental samples was conducted. It was found that the expression of PASK (^∗∗∗^*P* < 0.001) and SREBP-1c (^∗∗∗^*P* < 0.001) was increased in OA group compared with the control, while the level of PASK incubated with nuciferine (10, 25, and 50 μM, ^∗∗^*P* < 0.01 and ^∗∗∗^*P* < 0.001 vs. OA) and siRNA PASK (^∗∗∗^*P* < 0.001 vs. OA) was decreased compared with OA group. Similar results were obtained for SREBP-1c expression (^∗∗^*P* < 0.01 and ^∗∗∗^*P* < 0.001 vs. OA). Meanwhile, the expression of AMPK was decreased in OA group compared with control (^∗∗∗^*P* < 0.001 vs. control), and significantly increased in nuciferine (^∗∗∗^*P* < 0.001 vs. OA) and siRNA PASK groups (^∗∗∗^*P* < 0.001 vs. OA) (**Figures 6A,B**). Similar consequences were obtained in immunostaining assay (**Figure 6E**). In addition, to further evaluate the activation of AMPK, we have also measured the p-AMPK with p-AMPK antibody by western blot, finding that treatment of OA (^∗∗∗^*P* < 0.001 vs. control) downregulated the expression of p-AMPK, and this decreased level could be reversed by nuciferine and siRNA PASK (^∗∗∗^*P* < 0.001 vs. OA) (**Figure 6C**). Meanwhile, similar trend were emerged in the ratio of p-AMPK/AMPK (p-AMPK^∗^GAPDH(A)/GAPDH(p)^∗^AMPK) (^∗^*P* < 0.05) (**Figure 6D**), indicating that nuciferine could upregulate both the expression and activation of AMPK in OA-induced HepG_2_ cells. These results imply that nuciferine could regulate the expression of PASK, AMPK, SREBP-1c and relevant target genes during its inhibition of excessive lipogenesis.

### The Effect of Nuciferine or siRNA PASK on Cell Lipid Peroxidation and Oxidative Stress in OA-induced Hepatic Steatosis

**FIGURE 7:**
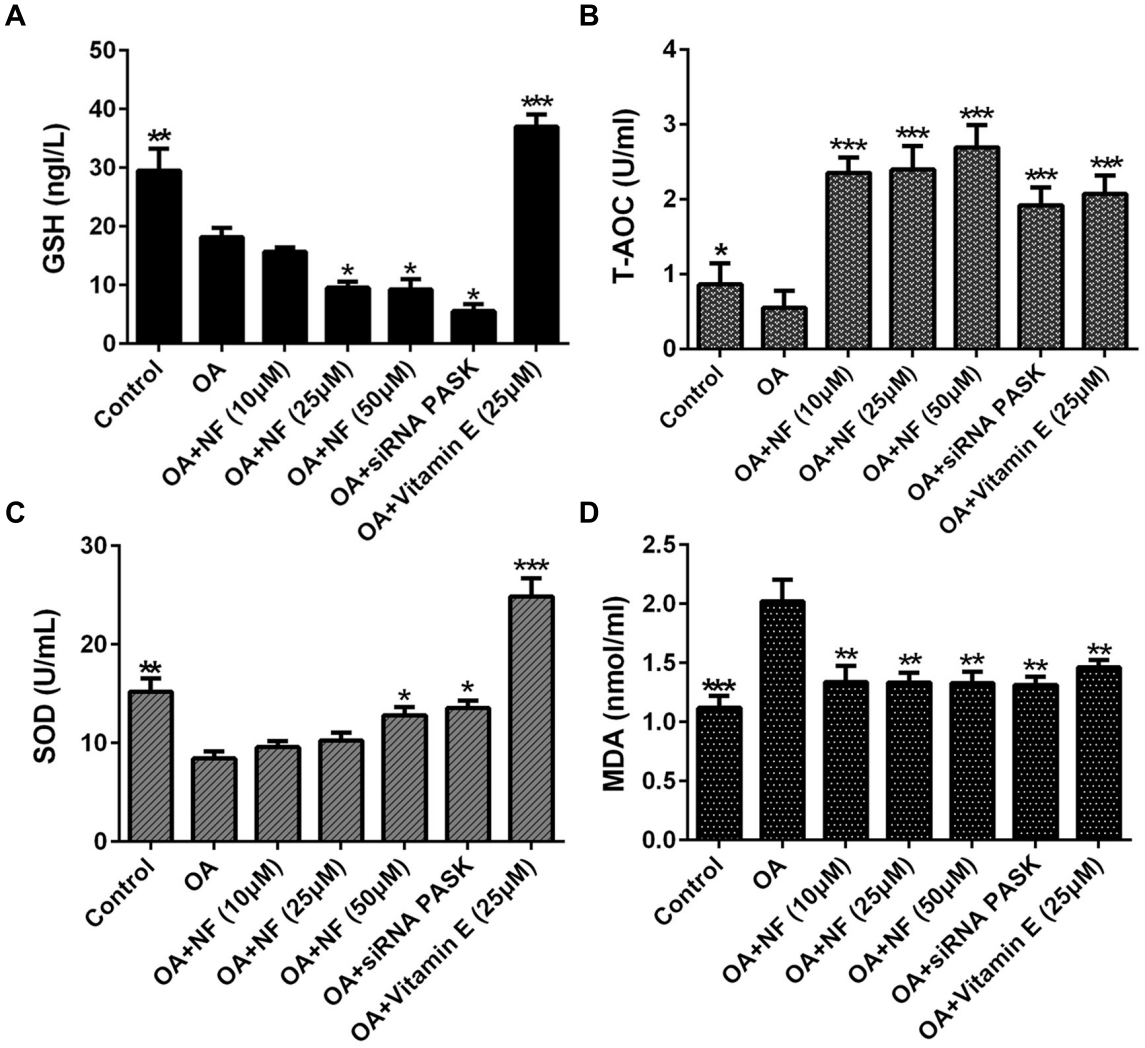
**The effect of nuciferine or siRNA PASK on cell lipid peroxidation in HepG_2_ cells of OA-induced hepatic steatosis. (A)** GSH. **(B)** T-AOC. **(C)** SOD. **(D)** MDA. SiRNA PASK HepG_2_ cells and HepG_2_ cells incubated with increased concentrations of nuciferine (NF: 10, 25, and 50 μM) or vitamin E (25 μM) both treated with OA (40 μM). The measurement was described in the Section “Materials and Methods”. Values are Mean ± SEM of three independent experiments performed in triplicates. Significant differences with OA group were designated as ^∗^*P* < 0.05, ^∗∗^*P* < 0.01, and ^∗∗∗^*P* < 0.001. (Control, siRNA scrambled; NF, Nuciferine.)

To see whether nuciferine or siRNA PASK could ameliorate lipid peroxidation and oxidative stress, the main factors contributed to NASH, we tested the content of GSH, T-AOC and SOD, which were selected as measurement of lipid peroxidation. The GSH level in OA group was significantly depleted compared with the control (^∗∗^*P* < 0.01). Upon addition of nuciferine, the content of GSH was surprising decreased by 13.51, 46.66 and 49% (^∗^*P* < 0.05 vs. OA), and siRNA PASK depleted by 69.97% (^∗^*P* < 0.05 vs. OA), but vitamin E significantly increased GSH level by twofold compared with the OA group (^∗∗∗^*P* < 0.001 vs. OA) (**Figure 7A**). The T-AOC and SOD level in OA group was significantly depleted compared with the control (^∗^*P* < 0.05 and ^∗∗^*P* < 0.01), and addition of nuciferine could simultaneously reverse the decrease level of T-AOC (^∗∗∗^*P* < 0.001 vs. OA) and SOD (^∗^*P* < 0.05 vs. OA), while the siRNA PASK (^∗∗∗^*P* < 0.001 and ^∗^*P* < 0.05 vs. OA) and vitamin E (^∗∗∗^*P* < 0.001 vs. OA) showed similar trend (**Figures 7B,C**). In addition, MDA was regarded as measurement of oxidative stress. The OA treated group could significantly increase MDA content compared with the control (^∗∗∗^*P* < 0.001). Addition of nuciferine diminished MDA level by 33.57, 34.07 and 34.12 (^∗∗^*P* < 0.01 vs. OA), and vitamin E (25 μM) and siRNA PASK were inhibited by 27.62% (^∗∗^*P* < 0.01 vs. OA) and 35% (^∗∗^*P* < 0.01 vs. OA) compared with the OA group (**Figure 7D**). These findings demonstrate that nuciferine and siRNA PASK could both successfully decrease the oxidative stress, probably owing to the down-regulation of antioxidant molecules SOD, T-AOC, but not GSH.

### The Effect of Nuciferine or siRNA PASK on Inflammatory Cytokines in OA-induced Hepatic Steatosis

**FIGURE 8:**
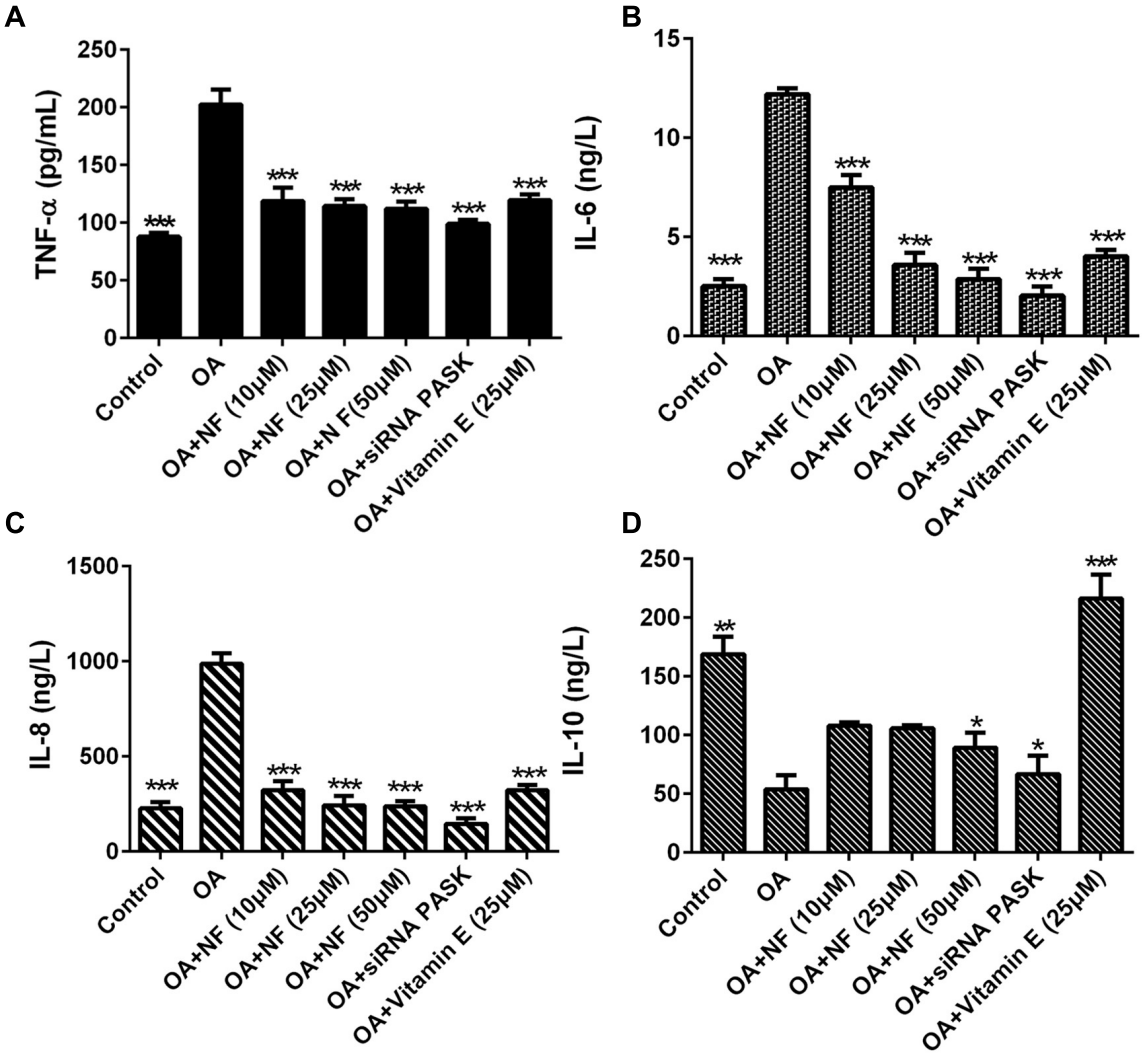
**The effect of nuciferine or siRNA PASK on inflammatory cytokines in HepG_2_ cells of OA-induced hepatic steatosis. (A)** TNF-α. **(B)** IL-6. **(C)** IL-8. **(D)** IL-10. SiRNA PASK HepG_2_ cells and HepG_2_ cells incubated with increased concentrations of nuciferine (NF: 10, 25, and 50 μM) or vitamin E (25 μM) both treated with OA (40 μM). The measurement was described in the Section “Materials and Methods”. Values are Mean ± SEM of three independent experiments performed in triplicates. Significant differences with OA group were designated as ^∗^*P* < 0.05, ^∗∗^*P* < 0.01, and ^∗∗∗^*P* < 0.001. (Control, siRNA scrambled; NF, Nuciferine.)

To clarify the possible role of nuciferine or siRNA PASK on inflammation, inflammatory cytokine levels were observed in OA induced HepG_2_ cells. It was found that the concentration of TNF-α increased by 2.3-fold (^∗∗∗^*P* < 0.001), IL-6 by 4.84-fold (^∗∗∗^*P* < 0.001), and IL-8 by 4.35-fold (^∗∗∗^*P* < 0.001) in OA-induced HepG_2_ cells as compared with the control. Nuciferine (10, 25, and 50 μM) decreased the level of TNF-α by 41.39, 43.45, and 44.57% (^∗∗∗^*P* < 0.001 vs. OA), IL-6 by 38.66, 70.53, and 76.53% (^∗∗∗^*P* < 0.001 vs. OA), and IL-8 by 67.31, 75.33, and 76.04% respectively (^∗∗∗^*P* < 0.001 vs. OA), while vitamin E and siRNA PASK also reverted the TNF-α increase by 40.84% (^∗∗∗^*P* < 0.001 vs. OA) and 51.08% (^∗∗∗^*P* < 0. 001 vs. OA), IL-6 by 67.08% (^∗∗∗^*P* < 0.001 vs. OA) and 83.29% (^∗∗∗^*P* < 0.001 vs. OA), and IL-8 by 67.41% (^∗∗∗^*P* < 0.001 vs. OA) and 85.2% (^∗∗∗^*P* < 0.001 vs. OA) compared with OA group (**Figures 8A–C**). In addition, IL-10 in OA treated cells was inhibited by 68.09% compared with the control (^∗∗^*P* < 0.01). Addition of nuciferine (10, 25, and 50 μM) increased the level of IL-10 by 2, 1.96, and 1.65-fold respectively (^∗^*P* < 0.05 vs. OA). Likewise, siRNA PASK and vitamin E (25 μM) improved the IL-10 by 1.24-fold (^∗^*P* < 0.05 vs. OA) and fourfold (^∗∗∗^*P* < 0.001 vs. OA) respectively compared with OA treated group (**Figure 8D**). These results imply that nuciferine and siRNA PASK may play a potential role in preventing and attenuating the process of inflammation, similarly.

### The Effect of Nuciferine or siRNA PASK on the Expression of Genes and Proteins Related to Inflammation

**FIGURE 9:**
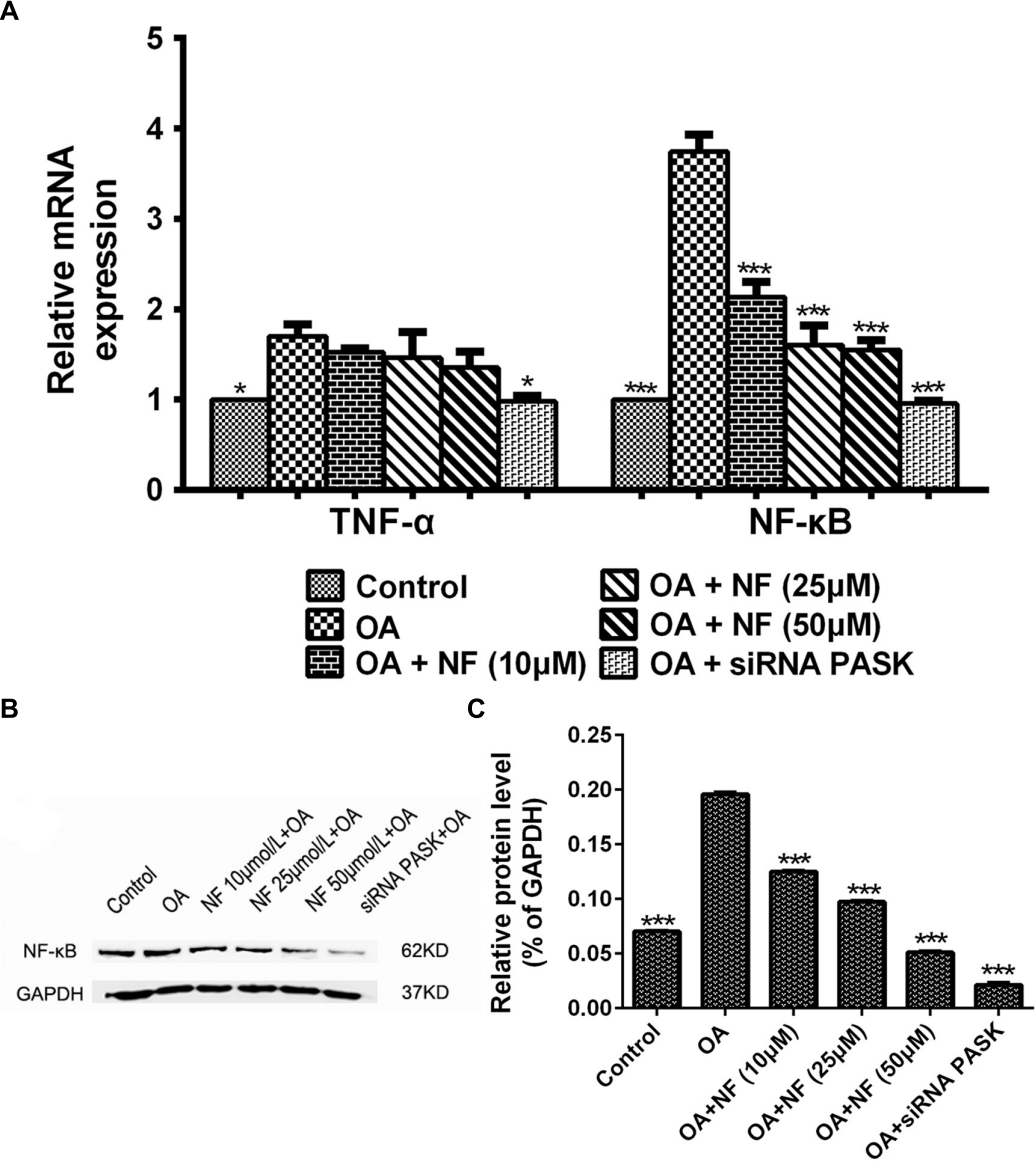
**(A)** The effect of nuciferine or siRNA PASK on the mRNA level related to inflammation in HepG_2_ cells. **(B,C)** The effect of nuciferine and siRNA PASK on the expression of proteins related to inflammation in HepG_2_ cells. SiRNA PASK HepG_2_ cells and HepG_2_ cells incubated with increased concentrations of nuciferine (NF: 10, 25, and 50 μM) both treated with OA (40 μM). The measurement was described in the Section “Materials and Methods”. Values are Mean ± SEM of three independent experiments performed in triplicates. Significant differences with OA group were designated as those ^∗^*P* < 0.05 and ^∗∗∗^*P* < 0.001. (Control, siRNA scrambled; NF, Nuciferine.)

To further deliberate the possible mechanism underlying the nuciferine-mediated effect on inflammation, the alteration of genes involved in release of inflammatory cytokines was measured. The expression of TNF-α (^∗^*P* < 0.05) and NF-κB (^∗∗∗^*P* < 0.001) mRNA in HepG_2_ cells treated with OA was increased significantly as compared with that in the control. Meanwhile, siRNA PASK could significantly inhibited the level of TNF-α (^∗^*P* < 0.05 vs. OA) and NF-κB (^∗∗∗^*P* < 0.001 vs. OA) mRNA compared with the OA-treated cells. However, addition of nuciferine (10, 25, and 50 μM) showed no statistically significant decrease in expression of TNF-α mRNA though it could significantly downregulate the NF-κB mRNA expression (^∗∗∗^*P* < 0.001 vs. OA) (**Figure 9A**). In addition, the expression of NF-κB (^∗∗∗^*P* < 0.001 vs. control) in HepG_2_ cells treated with OA was upregulated significantly as compared with the control, and this upregulation could be successfully reverted by nuciferine (10, 25, and 50 μM; ^∗∗∗^*P* < 0.001 vs. OA) and siRNA PASK (^∗∗∗^*P* < 0.001 vs. OA) (**Figures 9B,C**). These data suggest that inhibition of TNF-α and NF-κB might be the potential mechanism underlying the regulatory effect of nuciferine on inflammation, which was accompanied with downregulated expression of PASK.

## Discussion

Non-alcoholic fatty liver disease is often considered as the hepatic manifestation of the metabolic syndrome (MS) accompanied with a cluster of metabolic disorders including obesity, insulin resistance (IR), hypertension, and dyslipidemia (Tarantino et al., 2007). It is reported that approximate 80–90% obese adults in the western world suffer from NAFLD (Lazo and Clark, 2008; Bellentani et al., 2010). Currently, pervasive researches have been made to explore the effective therapy for NAFLD (Salamone et al., 2012b; Takahashi et al., 2015). Unlike vitamin E or other lipid-lowering drugs, nuciferine is an active ingredient extracted from the natural product lotus leaf (Xu et al., 2007). It is reported that nuciferine can exert a protective effect against liver steatosis and inflammation *in vivo* (Guo et al., 2013). As an emerging therapeutic agent for liver diseases, we speculate that nuciferine is a potential drug lead for the treatment of NAFLD.

As the mechanism underlying the regulatory effect of nuciferine on NAFLD remains uncertain, our studies used OA induced-HepG_2_ cells to establish liver steatosis models in an attempt to explore the specific mechanism *in vitro* (**Figure 1**). It was found in our study that the concentration of FFAs was increased significantly in OA-induced HepG_2_ cells (**Figure 4**), which resulted in lipotoxicity, increased TG content, lipid peroxidation, oxidative stress, and increased generation of inflammatory cytokines.

Per-Arnt-Sim kinase, as an emerging regulator on mammalian glucose and lipid metabolism, possesses a biological activity in the regulation of MS, and may exert a potential and pivotal impact on drug therapy of NAFLD. However, the effect mediated by PASK on NAFLD, especially on NASH, remains unknown. Thus, we silenced PASK in HepG_2_ cells and found that inhibition of PASK could reverse the events caused NAFLD/NASH, which was simultaneously evaluated along with nuciferine and vitamin E.

As a typical characteristic in the first stage of NAFLD, excessive stimulation (Ahmed et al., 2015) of hepatic lipogenesis resulted from the increased influx of FFAs into hepatocytes is a crucial risk factor leading to deterioration of NAFLD. Hence, attenuating or preventing hepatic lipogenesis is of great importance in treating NAFLD Guo et al. (2013) measured the concentration of TG and FFAs of hepatic tissue extracted from high-fat diet induced hamsters and found that nuciferine had a downregulated effect on lipid accumulation. Similarly, as shown in our study (**Figures 3** and **4**), nuciferine also involved in inhibition of lipid accumulation, independently and significantly reversing the concentration of TG and FFAs in OA-treated HepG_2_ cells without any other internal factors, which could eliminate other uncertain factors contributing to the catabatic role of nuciferine in decreasing hepatic lipid accumulation. Meanwhile, there was a decreased tendency of FFAs and TG in OA-treated siRNA PASK HepG_2_ cells, suggesting that PASK may involve in regulation of hepatic lipid accumulation, decreasing excessive lipogenesis. In addition, vitamin E was not as effective as nuciferine in reversing the biochemical parameters, although it also inhibited the stimulation of hepatic lipogenesis.

It has been proved that PASK is required for the proteolytic maturation of the SREBP-1c, and administration of a PASK inhibitor could downregulate hepatic expression of lipogenic SREBP-1c and its target genes, enhance mitochondrial respiration through upregulating the expression of AMPK pathway, decrease serum triglycerides, and partially reverse IR (Hao et al., 2007; Wu et al., 2014). Under such circumstances, to further decipher the possible mechanism of nuciferine that Guo et al. (2013) did not accomplish, we focused on PASK and evaluated the expression of lipogenesis-related genes including SREBP-1c, FAS, ACC, SCD1, lipid-responsive nuclear hormone receptor (PPARα and PPARγ) and further observe the alterations of two relevant target genes of PASK (AMPK and Akt) in nuciferine and siRNA PASK group. Interestingly, nuciferine decreased the expression of PASK and its relevant target genes PPARα, PPARγ, SREBP-1c, FAS, ACC, and SCD1, and upregulated the expression and activation of AMPK that regulates the mitochondrial respiration and lipid metabolism. In addition, the same alternations were observed in OA-treated siRNA PASK HepG_2_ cells (**Figure 5**), indicating that PASK participated in lipid metabolism, and therefore PASK might be a potential therapeutic target for ameliorating hepatic lipid accumulation, and even could be an effective treatment for NAFLD and other MS accompanied with lipid accumulation or obesity. More importantly, as compared with similar effects of PASK inhibition and nuciferine on alleviating lipogenesis, nuciferine could also downregulate the PASK expression and alter the level of relevant genes of PASK. Hence, our finding suggest for the first time that PASK-mediated inhibition of lipid accumulation may be a potential mechanism of nuciferine in alleviating NAFLD probably by regulating the expression of PASK, AMPK, SREBP-1c, and relevant targets genes (**Figure 6**). However, further studies are required to clarify whether the mediation of PASK on effect of nuciferine is predominant or dispensable.

Interestingly, we found an increased expression of Akt in nuciferine-treated steatotic cells and OA-treated siRNA PASK HepG_2_ cells compared with the depleted level in OA-induced HepG_2_ cells (**Figure 5**). The PI3K/Akt pathway is known to promote GLUT4 expression in myocytes and adipocytes (Bryant et al., 2002), and the activation of Akt may be an essential requirement for ameliorating IR, a typical characteristic of NAFLD/NASH (Vidyashankar et al., 2013; Hojlund, 2014). In accordance with earlier findings that nuciferine (Nguyen et al., 2012; Guo et al., 2013) and PASK (Fontes et al., 2009; Sabatini and Lynn, 2015) could effectively attenuate IR, our result found a increased expression of Akt in OA-treated cells incubated with nuciferine and siRNA PASK HepG_2_ cells, suggesting that PASK may play an important role in IR, and PASK inhibition might be part of mechanism of the nuciferine-mediated regulative effect on IR. However, further investigations are required to independently deliberate the specific mechanism of PASK in nuciferine-mediated treatment of IR.

Increased FFAs influx into HepG_2_ cells may cause severe lipotoxicity, leading to an imbalance between pro-oxidants and antioxidants (Abdul-Ghani et al., 2008), which is likely to increase the generation of reactive oxygen species (ROS) and MDA (Nourazarian et al., 2014) that induce a series of cellular damage (Hoffman and Brookes, 2009). Earlier studies (Guo et al., 2013) had proved that nuciferine could decrease the MDA in high-fat diet induced hamsters, but the cause remains inconclusive. Furthermore, whether PASK contributed to the reversed concentration of MDA mediated by nuciferine also remained unknown. Therefore, we similarly tested the increases of ROS-mediated lipid peroxidation by measuring the concentration of MDA from cell medium. It was found that the elevated MDA level induced by OA was significantly reversed by nuciferine administration in OA-treated HepG_2_ cells and OA-treated siRNA PASK cells (**Figure 7D**). Although vitamin E also showed the similar effect, it was not effective as nuciferine, confirming that nuciferine could attenuate lipid peroxidation by decreasing the concentration of MDA, which may be partly related to the downregulated expression of PASK. More importantly, our findings also first proved that PASK potentially plays a regulative role in mammalian [not yeast (Huang et al., 2014)] lipid peroxidation and oxidative stress, which may provide a reference for future research on physiological function of PASK.

Oxidative stress is caused by an imbalance in the redox status of the body (Nourazarian et al., 2014). Thus, maintaining the balance between pro-oxidant and antioxidant molecules is imperative to downregulate ROS level. As an antioxidant molecule, GSH plays a pivotal role in defending against ROS (Kedderis, 1996). Studies (Guo et al., 2013) have shown that nuciferine could downregulate the GSH *in vivo*. Surprisingly, our result showed that the depleted GSH levels in OA-induced group were not replenished in nuciferine treated OA-HepG_2_ cells and the OA-treated siRNA PASK HepG_2_ cells, while the level of GSH in vitamin E showed significant increased (**Figure 7A**). Although the reason is unknown, we speculate that the regulation on GSH *in vivo* may be due to other participants such as regulative enzymes or receptors but may not directly result from nuciferine. Meantime, the results also imply that PASK exerted no positive impact on GSH when the level of GSH in OA-treated siRNA PASK HepG_2_ cells was significantly decreased. However, the concentration of T-AOC (Wang et al., 2015a) and SOD (Kwon et al., 2012) were reversed by nuciferine and siRNA PASK respectively as well as vitamin E (**Figures 7B,C**), demonstrating that nuciferine could successfully decrease the oxidative stress probably owing to the down-regulation of antioxidant molecules SOD and T-AOC but not GSH, which was accompanied with the decreased expression of PASK. These results suggest that the inhibition of PASK may play an imperative role in downregulating oxidative stress controlled by nuciferine based on the similar results from nuciferine and siRNA PASK groups. Further comprehensive investigation on PASK participants in the regulation of pro-oxidants and antioxidants is required.

Besides accumulation of lipogenesis, lipotoxicity, lipid peroxidation, and oxidative stress, inflammation is another crucial factor in the pathogenesis of NAFLD that aggravates the progression from hepatocellular steatosis to NASH (Donath, 2013; Ahmed et al., 2015). Several previous studies (Guo et al., 2013; Wang et al., 2015b) indicated that nuciferine may be potential for the prevention and treatment of kidney inflammation or liver inflammation, but the mechanism is indeterminate. Thus, to determine the function of nuciferine on the release of inflammatory cytokines, we evaluated the TNF-α (Lin et al., 2015), IL-6 (Mykhal’chyshyn et al., 2013) in similar lines with earlier findings, and further observed the alterations of IL-8 and IL-10 (Burger, 2013; Fetahu et al., 2014). The results obtained in our study showed that TNF-α, IL-6 and IL-8 secretion were significantly increased by addition of OA paralleled with depleted IL-10 level. In contrast, decreased levels were observed when OA-induced HepG_2_ cells were incubated with nuciferine and vitamin E, and the IL-10 level showed significantly increased. These results further show that nuciferine could inhibit inflammation through blocking the release of TNF-α, IL-6 and IL-8 and promoting the IL-10 level. In addition, there is no evidence of a pronounced relationship between PASK and inflammation. To explore the possible effect of PASK on inflammation and the potential role of PASK in the nuciferine-mediated anti-inflammatory effect, we examined the alterations of TNF-α, IL-6, IL-8, and IL-10 in OA-induced siRNA PASK HepG_2_ cells in similar lines with nuciferine. Interestingly, the reversed effect of TNF-α, IL-6, IL-8, and IL-10 emerged in OA-induced siRNA PASK HepG_2_ cells, as well as in nuciferine and vitamin E (**Figure 8**). These findings demonstrate for the first time that PASK could efficiently attenuate inflammation by blocking the release of inflammation cytokines, suggesting that PASK might emerge as a new drug target for inflammation such as NASH due to its anti-inflammatory effect, which was consistent with effects of nuciferine. In addition, it was reported that the NF-κB pathway (Okamoto et al., 2007) played an important role in contributing to the NAFLD pathogenic process, and may be a possible target for alleviation of NASH (Salamone et al., 2012a). Therefore, we evaluated the alterations of NF-κB and TNF-α on gene and protein levels in order to decipher the possible mechanism of siRNA PASK and nuciferine mediated anti-inflammation. It was found that OA treatment significantly elevated the expression of NF-κB and TNF-α in the liver. However, treatment with nuciferine or vitamin E blocked OA-induced expression of NF-κB and TNF-α, which is consistent with the results in OA-induced siRNA PASK HepG_2_ cells (**Figure 9**). Therefore, according to the results that both nuciferine and siRNA PASK could alleviate inflammation by preventing the release of inflammation cytokines such as TNF-α, IL-6, and IL-8, and nuciferine could downregulate the expression of PASK, TNF-α, and NF-κB, which was similar to the alteration in siRNA PASK group, taken together, we speculate the inhibition of PASK may be predominate or part of the mechanism underlying the anti-inflammation function of nuciferine. These findings may be preliminary, and our future research will further address the specific role of PASK in nuciferine-mediated anti-inflammatory effect.

## Conclusion

Our results demonstrate that nuciferine possesses potent function on attenuating or inhibiting lipid accumulation and inflammation and regulates the expression of PASK and its relevant target genes involved in lipid metabolism and inflammation in OA-induced HepG_2_ cells, indicating that nuciferine and PASK have a potential inhibitory effect against NAFLD/NASH, and the PASK might play an important role in nuciferine-mediated regulation. Our future investigations will be dedicated to additional *in vivo* work.

## Author Contributions

The author’s contributions are as follows: D-DZ and X-YL designed the study. D-DZ, and J-GZ performed all experiments with cells and wrote this article. YL and Y-ZW consult and categorize related references. XW, S-YG, and G-HZ supervised the data analysis. X-YL and G-LL supervised the research. All authors contributed to and approved the final version of the manuscript.

## Conflict of Interest Statement

The authors declare that the research was conducted in the absence of any commercial or financial relationships that could be construed as a potential conflict of interest.
